# Lichen-Associated Fungal Community in *Hypogymnia hypotrypa* (Parmeliaceae, Ascomycota) Affected by Geographic Distribution and Altitude

**DOI:** 10.3389/fmicb.2016.01231

**Published:** 2016-08-05

**Authors:** Yanyan Wang, Yong Zheng, Xinyu Wang, Xinli Wei, Jiangchun Wei

**Affiliations:** ^1^State Key Laboratory of Mycology, Institute of Microbiology – Chinese Academy of SciencesBeijing, China; ^2^Key Laboratory for Plant Diversity and Biogeography of East Asia, Kunming Institute of Botany – Chinese Academy of SciencesKunming, China

**Keywords:** lichens, endolichenic fungi, lichenicolous fungi, fungal community, 18S rRNA gene, Tibetan Plateau

## Abstract

Lichen-associated fungal species have already been investigated in almost all the main growth forms of lichens, however, whether or not they are homogeneous and constant within each lichen species are still inconclusive. Moreover, the related ecological factors to affect and structure the fungal composition have been poorly studied. In order to answer these questions, we took *Hypogymnia hypotrypa* as a model to study the relationship between the lichen-associated fungal composition and two ecological factors, i.e., site and altitude, using the method of IlluminaMiSeq sequencing. Four different sites and two levels of altitude were included in this study, and the effects of site and altitude on fungal community composition were assessed at three levels, i.e., operational taxonomic unit (OTU), class and phylum. The results showed that a total of 50 OTUs were identified and distributed in 4 phyla, 13 classes, and 20 orders. The lichen-associated fungal composition within *H. hypotrypa* were significantly affected by both site and altitude at OTU and class levels, while at the phylum level, it was only affected by altitude. While the lichen associated fungal communities were reported to be similar with endophytic fungi of the moss, our results indicated the opposite results in some degree. But whether there exist specific OTUs within this lichen species corresponding to different sites and altitudes is still open. More lichen species and ecological factors would be taken into the integrated analyses to address these knowledge gaps in the near future.

## Introduction

Lichen-associated fungi are composed of endolichenic and lichenicolous fungi. Comparing with endophytes, which are organisms that live inside other organisms without producing any apparent disease symptoms (Strobel and Daisy, 2003), endolichenic fungi refers to the endophytes isolated from lichens (symbiont of fungi and algae and/or cyanobacteria), and lichenicolous fungi are those living exclusively on the lichen thalli. These are distinct from lichen mycobionts (the symbiotic fungal component of lichen thallus) and lichenicolous fungi (Lawrey and Diederich, 2003; Arnold et al., 2009). Almost all the main growth forms of lichens have been studied in order to approach the diversity of lichen-associated fungi, such as crustose lichen species *Acarospora fuscata* (Nyl.) Th. Fr., *Lecanora* spp. (Muggia and Grube, 2010) and *Caloplaca* spp., *Lecidella* spp. (Harutyunyan et al., 2008); foliose lichen species *Dermatocarpon miniatum* (L.) W. Mann, *Physconia americana* Esslinger, *Physcia dimidiata* (Arnold) Nyl. (Harutyunyan et al., 2008), *Lobaria* spp., *Peltigera* spp., *Umbilicaria mammulata* (Ach.) Tuck. (Arnold et al., 2009), *Parmelia sulcata* Taylor (Muggia and Grube, 2010), *Heterodermia* spp. and *Parmotrema* spp. (Tripathi et al., 2014); and fruticose lichen species *Cladonia* spp. (Muggia and Grube, 2010; U’Ren et al., 2012), *Pseudevernia intensa* (Nyl.) Hale & W. L. Culb. and *Usnea hirta* (L.) Weber ex F. H. Wigg. (U’Ren et al., 2010).

Three points could be concluded through the previous publications: firstly, two methods were applied to study the lichen-associated fungi, i.e., culture-based and culture-free (next-generation sequencing) methods, in which the diversity reflected by the former was much less than by the latter (Arnold et al., 2007; Higgins et al., 2011; Bálint et al., 2013); secondly, the lichen-related fungi are mainly composed of members of Ascomycota (Dothideomycetes, Eurotiomycetes, Leotiomycetes, Pezizomycetes, and Sordariomycetes, etc.) and Basidiomycota (Agaricomycetes and Tremellomycetes, etc.; U’Ren et al., 2010, 2012, 2014); thirdly, relative to the endophytes in the living and dead photosynthetic tissues of plants, the lichen-related fungi are largely distinctive (U’Ren et al., 2010, 2012; Zhang et al., 2015), whereas similar to endophytes of mosses (U’Ren et al., 2010, 2012). Although, some lichen-related fungi are specialized to some certain lichen species (Tripathi et al., 2014), and compositions of lichen-related fungal communities have been shown to differ among host taxa (U’Ren et al., 2010), the lichen-related fungi are common in lichens with different substrates (Li et al., 2007; Kannangara et al., 2009).

The factors shaping the structure of endophytes or lichen-related fungal communities have not been well-studied. Some studies attributed those to abiotic factors (Lau et al., 2013), such as climate (U’Ren et al., 2012), exposure and season (Beck et al., 2014), and geographic distance (Higgins et al., 2014; Zhang et al., 2015) as well. The latest research showed that interaction type was more important than the local abiotic conditions (Chagnon et al., 2016). Most of the above studies relied on the analysis to the fungal cultures, and even the available researches on lichen-related fungi by culture-independent approach (e.g., pyrosequencing), however, didn’t concern the potential shaping ecological factors in natural ecosystems. Therefore, it’s highly necessary to address how the biotic or abiotic factors affect the lichen-related fungal community composition using molecular techniques, such as the pyrosequencing.

*Hypogymnia hypotrypa* (Nyl.) Rassad. is an interesting species, which belongs to one of the largest family of lichen-Parmeliaceae (Ascomycota), and has peculiar morphological characters: swollen lobes, hollow medulla, and perforations at the lower surface (McCune and Obermayer, 2001). Besides, there is a rather wide distribution and broad altitude range of *H. hypotrypa* in modern China. Thus, in this study, *H. hypotrypa* is chosen as the model in order to investigate the lichen-associated fungal community. Specifically, we ask the following questions: (1) Are there host-specific fungal species in *H. hypotrypa* and what are the differences in fungal composition between *H. hypotrypa* and other lichen species? (2) Do the lichen-associated fungal community have constant composition within *H. hypotrypa*? and do any related ecological factors exist to affect and structure fungal composition?

## Materials and Methods

### Reagents

The main reagents and kits used in this study were CTAB and PCR products purification kit. CTAB used for DNA extraction contains 2% CTAB (W/V), 1.4 M NaCl, 20 mM EDTA, and 10 mM Tris-HCl (pH 8.0). PCR products purification kit produced by Axygen Biosciences, Inc., USA, includes Buffer DE-A, Buffer DE-B, Buffer W1, Buffer W2 concentrate and Eluent (2.5 mM Tris-HCl, pH 8.5).

### Site Descriptions and Sampling

Four sites were chosen according to the main distribution of Chinese *H. hypotrypa*, including Shaanxi Province, Sichuan Province, Tibet, and Yunnan Province, among which the first site situates in central China, and the others are located in southwestern China. The specimens were identified based on the morphology and three-locus DNA sequences, i.e., ITS (internal transcribed spacer), GPDH (glycerol-3-phosphate dehydrogenase), and MCM7 (DNA replication licensing factor). Taking the altitude 3500 m as boundary, middle altitude (M) was named to the site less than 3500 m, and high altitude (H) to the site more than 3500 m. There are two types of altitude in Tibet, and all the other sites only have one type, respectively. At each site, three specimens of *H. hypotrypa* in duplicates were sampled, so 15 lichen samples were included and shown in **Table 1**. The lichen samples were frozen at –80°C not more than 1 month until DNAs were extracted.

**Table 1:** The collection information of 15 lichen samples in this study.

Sample	Collection information	Site type	Altitude (m)	Altitude type
SC-H-1	Sichuan, 2014.8.23, H. Li & S. H. Jiang SC201408834	A	4190	H
SC-H-2	Sichuan, 2014.8.23, H. Li & S. H. Jiang SC201408835	A	4190	H
SC-H-3	Sichuan, 2014.8.23, H. Li & S. H. Jiang SC201408833	A	4190	H
SX-M-1	Shaanxi, 2014.9.2, X. Y. Wang	B	3130	M
SX-M-2	Shaanxi, 2014.8.29, X. Y. Wang	B	3000	M
SX-M-3	Shaanxi, 2014.9.1, X. Y. Wang	B	3300	M
XZ-H-1	Tibet, 2014.9.7, X. L. Wei & Y. Y. Wang XZ20140101	C	3885	H
XZ-H-2	Tibet, 2014.9.7, X. L. Wei & Y. Y. Wang XZ20140833	C	3876	H
XZ-H-3	Tibet, 2014.9.7, X. L. Wei & Y. Y. Wang XZ20140834	C	3876	H
XZ-M-1	Tibet, 2014.9.7, X. L. Wei & Y. Y. Wang XZ2014237	C	3258	M
XZ-M-2	Tibet, 2014.9.7, X. L. Wei & Y. Y. Wang XZ2014235	C	3258	M
XZ-M-3	Tibet, 2014.9.7, X. L. Wei & Y. Y. Wang XZ2014244	C	3258	M
YN-M-1	Yunnan, 2014.8.17, H. Li & S. H. Jiang YN201408266	D	3281	M
YN-M-2	Yunnan, 2014.8.17, S. H. Jiang & H. Li YN201408271	D	3281	M
YN-M-3	Yunnan, 2014.8.17, S. H. Jiang & H. Li YN201408258	D	3281	M

### DNA Extraction

Prior to DNA extraction, 15 healthy foliose branches were chosen and surface cleaned. Lichen samples were surface sterilized by immersion in 75% ethanol for 1 min, in 1% sodium hypochlorite for 2 min, and in 75% ethanol for 0.5 min. The surface sterilized tissues were then rinsed with sterile water for 0.5 min and blotted dry with sterile filter paper. Each branch of lichen samples was cut into about 10 small fragments (1–2 cm long). The extraction procedure followed the modified CTAB method (Rogers and Bendich, 1985). The genomic DNA were examined by 0.8% AGE (agarose gel electrophoresis) and then the satisfactory DNA extracts were processed the subsequent PCR and sequencing.

### IlluminaMiSeq Sequencing

The fungal 18S rDNA region was amplified using the primers 0817F (5′-TTAGCATGGAATAATRRAATAGGA-3′) and 1196R (5′-TCTGGACCTGGTGAGTTTCC-3′; Rousk et al., 2010). The 20 μl reaction volume contained 10 ng template DNA, 0.6 μl each primer (5 μM), 4 μl 5× FastPfu buffer, 2 μl dNTPs (2.5 mM), 0.4 μl FastPfu polymerase and ultra-pure sterilized water (ddH_2_O). PCR amplifications were carried out in an ABI GeneAmp^®^ 9700 type PCR machine, following conditions: an initial denaturation at 95°C for 2 min, 32 cycles of denaturation at 95°C for 45 s, annealing at 55°C for 45 s, and extension at 72°C for 45 s, with a final extension at 72°C for 10 min. Each DNA extract was PCR amplified by three replicates, which were mixed into one PCR product, examined by 2% agarose gel, and then purified by AxyPrepDNAGel Extraction Kit (Axygen Biosciences, Inc., USA). The purified PCR products were quantificationally examined by QuantiFluor^TM^ -ST (Promega Corporation, USA) quantitative system, and the equimolar mixtures of multiple amplicons were obtained. After that, the MiSeq library was constructed and pyrosequencing were processed on an IlluminaMiSeq sequencing platform (Illumina, USA) at Majorbio Bio-Pharm Technology, Co., Ltd, Shanghai, China. The raw sequence reads were deposited in the NCBI sequencing read archive under Accession No SRP068130.

### Pyrosequencing Data Treatment

Raw reads were trimmed for adaptor sequence and for quality control using a sliding window approach implemented in Trimmomatic (Bolger et al., 2014). Overlapping paired-end reads were merged using Flash software (Magoc and Salzberg, 2011), the minimum overlap is set to 10 bp, and other parameters were at default settings. Lastly, the quality-checked sequences were clustered into operational taxonomic units (OTUs) using Usearch program version 7.1^1^ with a cut-off of 97% sequence identity (Edgar, 2013).

Taxonomy assignment was done using the Ribosomal Data base Project (RDP) classifier (Wang et al., 2007) implemented in Quantitative Insights Into Microbial Ecology (QIIME), sequences representing the OTUs were subjected to BLASTn searches in SILVA (Quast et al., 2013) databases (version 119) and GenBank, the corresponding accession No in GenBank of the similar sequence to each OTU was provided.

### Statistical Analyses

We executed a permutational multivariate analysis of variance (PERMANOVA; Anderson, 2001) analysis using the “adonis” function of the Vegan package with 999 permutations in R (Version 3.1.2) to test the effects of site and altitude as two factors on the community structure of lichen-associated fungi. The datasets of lichen-associated fungal total OTUs, Class and Phylum were normalized using the relative abundance transformation prior to all PERMANOVA analysis. The indicator OTU analyses were performed in order to locate the OTUs, classes and phylum with the most obvious response to site and altitude. Then in order to remove reciprocal effect of each factor, *t*-test and one-way ANOVA were made in this study.

Based on the OTU composition, the alpha-diversity and beta-diversity metrics were calculated. The α-diversity indices, Shannon and Simpson, were calculated using MOTHUR version 1.30.1 (Schloss et al., 2011). The β-diversity analysis, a principal coordinate analysis (PCoA), was used to visualize the effects of two factors on community composition of five set samples conducted in R. In addition, in order to compare and clarify the composition pattern of OTUs in the five set samples, gehpi diagram was also drawn using Gephi 0.8.2 software.

## Results

### Sequence Data

The raw data from 15 samples consisted of 250,182×2 reads and a total base is 125,591,364 bp, after removing the adaptor sequence and quality-control, 94,639,764 bp were obtained, of which 99.16% were longer than 401 bp. For each sample, the trimmed sequences and average length were displayed in Supplementary Table S1. After quality filtering, de-replication and singletons exclusion, the reads were clustered into 64 OTUs at a 97% identity threshold. Removing the host lichen-forming fungal OTU, 50 fungal OTUs of 26,708 reads were included in the final matrix (Supplementary Tables S1–S3). The number of OTUs in the 15 samples ranged from 24 to 33 (**Table 2**).

**Table 2:** The alpha diversity indexes of OTUs in the 15 lichen samples.

Sample	OTU no	Shannon index	Simpson index
SC-H-1	26	2.159218	0.838387
SC-H-2	29	2.435747	0.872458
SC-H-3	31	2.343006	0.86691
SX-M-1	24	2.164194	0.864683
SX-M-2	29	2.119863	0.841378
SX-M-3	25	2.040855	0.829514
XZ-H-1	25	2.300726	0.853435
XZ-H-2	24	2.102592	0.772671
XZ-H-3	29	1.447568	0.590744
XZ-M-1	31	2.462237	0.8803
XZ-M-2	28	2.302912	0.863782
XZ-M-3	33	2.30973	0.846609
YN-M-1	27	2.471464	0.890976
YN-M-2	29	2.507511	0.884155
YN-M-3	31	2.440719	0.883757

### Fungal Community Structure

**FIGURE 1:**
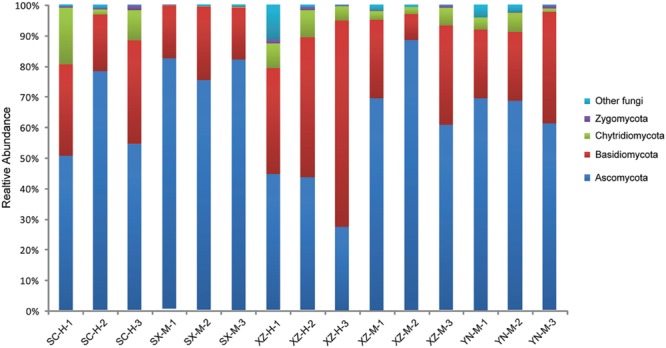
**The fungal community structure at the phylum level in the 15 samples.** The histogram was created using EXCEL and processed using Adobe Illustrator CS4. Different colors represent the different phyla of fungi.

**FIGURE 2:**
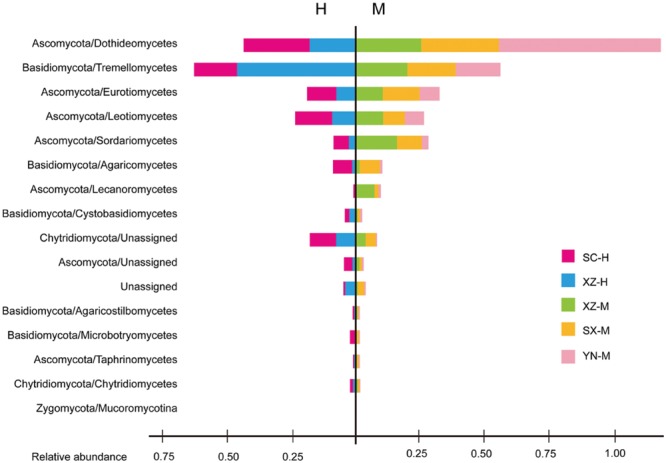
**The fungal community structure at the class level in the five set samples.** The histogram was created using EXCEL and processed using Adobe Illustrator CS4. Two parts are cut by the vertical line to separate the groups with two types of altitude (H and M). Different colors represent the different groups of samples, including SC-H, XZ-H, XZ-M, SX-M, and YN-M.

The taxonomic information and distribution of the 50 OTUs were presented in Supplementary Table S3. Among these OTUs, 28 belonged to Ascomycota, 11 to Basidiomycota, 4 to Zygomycota, 3 to Chytridiomycota, and 4 to unknown fungi (**Figure 1**). Furthermore, these fungal OTUs spanned 13 classes, 23 orders, 29 families and 25 known genera, among which the number of genera was less than families due to the limitations of DNA sequences identity. Within Ascomycota, four major classes (Dothideomycetes, Eurotiomycetes, Leotiomycetes, and Sordariomycetes), and 14 orders were included, among which Capnodiales was the most abundant, followed by Helotiales, Chaetothyriales, Hypocreales, and Pleosporales. The Basidiomycota contained one major class (Tremellomycetes) and six orders, with Tremellales being the most abundant (**Figure 2**).

### Dissimilarity of Fungal Communities among Different Lichen Individuals

The Shannon and Simpson’s indices were used to evaluate and compare the diversity of the fungal communities among the 15 samples (**Table 2**). The Shannon’s Index (1.44–2.50), also considering the Simpson’s values, and distribution of the 50 OTUs in the 15 samples, showed that the composition of fungal communities among the 15 samples were not very similar, especially the sample XZ-H-3 was more obviously different from others (**Figure 1**).

### Effect of Site on Composition of Fungal Communities at Different Levels

**FIGURE 3:**
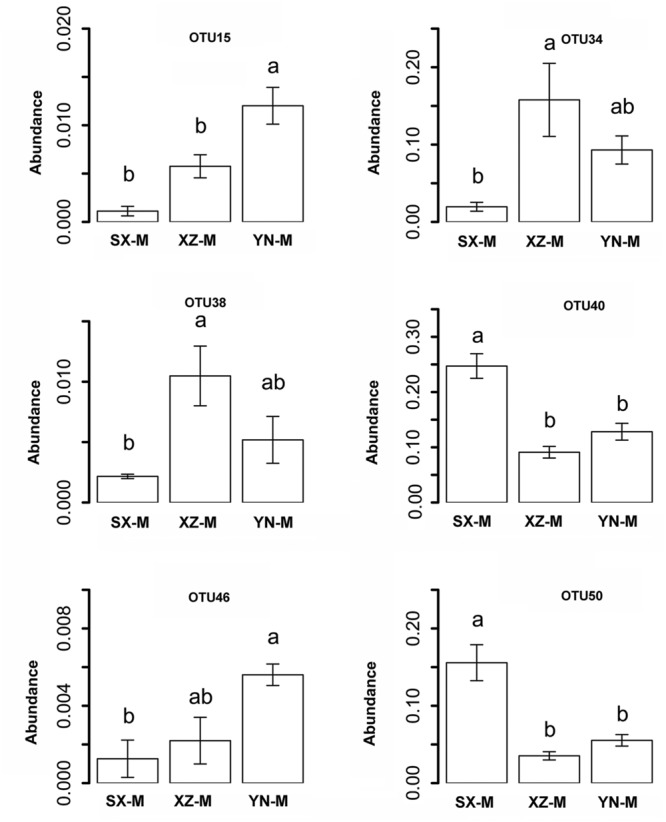
**The relative abundance of indicator OTUs with site effect.** The error bars indicate the standard error (mean ± SE). Different letters above the bars indicate the significance under *P* < 0.05. SX-M, XZ-M, and YN-M represent the three localities with same type of altitude.

**FIGURE 4:**
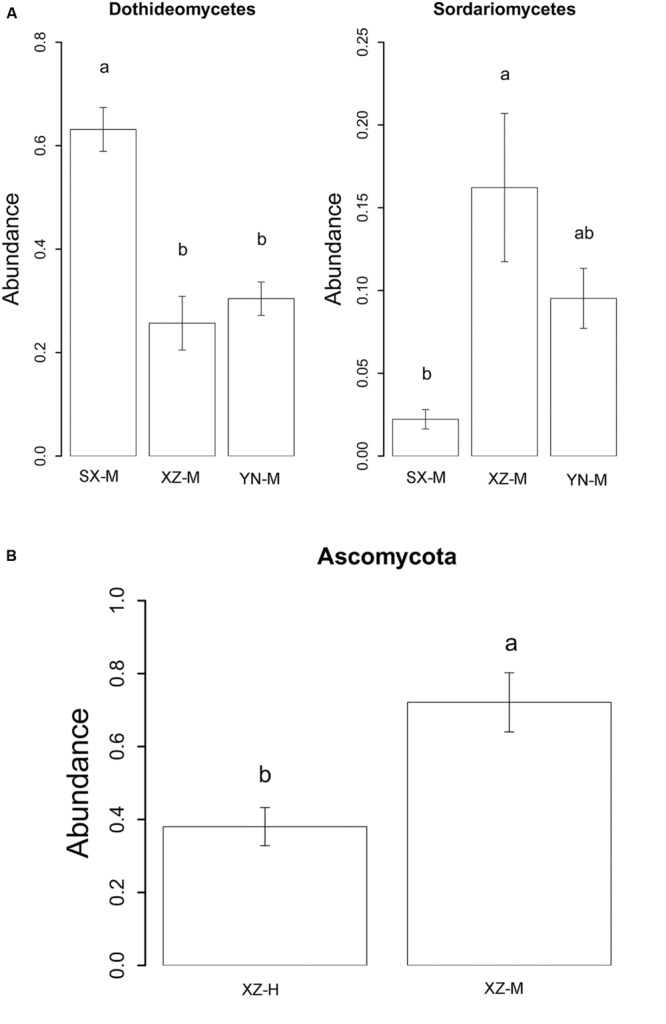
**The relative abundance of indicator class (A) with site effect and phylum (B) with altitude effect.** The histogram was graphed using R. The error bars indicate the standard error (mean ± SE). Different letters above the bars indicate the significance under *P* < 0.05. SX-M, XZ-M, and YN-M represent the three localities with same type of altitude. XZ-H and XZ-M represent the two altitudes of same locality.

The effect of site on composition of fungal communities at three levels, i.e., OTU, class, phylum, were evaluated based on the relative abundance data (Supplementary Tables S4–S6). Two factors PERMANOVA analyses were performed (**Table 3**). Four sites were grouped in this analysis, including Sichuan (SC-H, A), Shaanxi (SX-M, B), Tibet (XZ-H and XZ-M, C), and Yunnan (YN-M, D). The results showed that the fungal composition in the 15 samples of *H. hypotrypa* had significant difference at OTU level (**Table 3**, *P* = 0.001 < 0.01) and at class level (**Table 3**, *P* = 0.001 < 0.01), however, not at phylum level (**Table 3**, *P* = 0.08 > 0.05). Because there were two types of altitude (H and M) mixed in the site analysis, and in order to remove the reciprocal effect brought by altitude, the indicator OTU analysis being aimed at site, was carried out in R. It indicated that four OTUs (OTU15, OTU40, OTU46, and OTU50), six classes (Agaricomycetes, Agaricostilbomycetes, Lecanoromycetes, Sordariomycetes, Tremellomycetes, and Unassigned), and one phylum (Zygomycota) had site effect (Supplementary Table S7, all the *P* < 0.05). Then one-way ANOVA (among three treatments) were performed to disclose whether there existed differences among different treatment. The result supported that six OTUs (OTU15, OTU34, OTU38, OTU40, OTU46, and OTU50) and two classes (Dothideomycetes and Sordariomycetes) had significant differences among three sites with same level of altitude (M) (**Table 4**; **Figures 3** and **4A**).

**Table 3:** Two factors permutational multivariate analysis of variance (PERMANOVA) analysis on the site and altitude of all samples at OTU, class and phylum levels.

Ecological factors	Df	OTU level		Class level		Phylum level	
		F	P	F	P	F	P
Site (S)	3	2.94 0.001***		3.30 0.001***		2.79 0.080	
Altitude (A)	1	2.51 0.028*		3.19 0.012*		9.54 0.001***	

Significant codes. *0.01 < P < 0.05; **0.001 < P < 0.01; ***P < 0.001.

**Table 4:** The one-way ANOVA result for the indicator phylum, class and OTU with site effect (SX-M vs. XZ-M vs. YN-M).

Indicator	Indicator value	Probability	*t*-test or one-way ANOVA *P*-value
Ascomycota_Dothideomycetes	–	–	0.037
Ascomycota_Sordariomycetes	0.45	0.038	0.006
OTU15	0.50	0.026	0.003
OTU34	–	–	0.044
OTU38	–	–	0.046
OTU40	0.43	0.040	0.001
OTU46	0.51	0.035	0.039
OTU50	0.48	0.031	0.002

*Indicator value ≥0.25 were considered importantly indicative as suggested by Dufrene and Legendre (1997).*

### Effect of Altitude on Composition of Fungal Communities at Different Levels

The effect of altitude on composition of fungal communities at the same three levels was evaluated. The fungal composition structure was seen in **Figure 2**. It can be seen that the ratio of Ascomycota/Basidiomycota was higher in the medium-altitude (M) samples than in the high-altitude (H) samples, and at class level, there was also rather obvious difference in fungal composition between M and H samples. Two factors PERMANOVA analyses were performed (**Table 3**). Two types of altitude were included in this analysis (H and M). The results showed that the fungal composition in the 15 samples of *H. hypotrypa* had significant difference at OTU level (**Table 3**, *P* = 0.028 < 0.05), class level (*P* = 0.012 < 0.05), and phylum level (*P* = 0.001 < 0.01). Because there was site factor mixed in the altitude analysis, and in order to remove the reciprocal effect brought by site, the indicator OTU analysis being aimed at altitude, was carried out in R. The results indicated that there was not any indicator OTU, class and phylum being found in two altitudes (H and M) of same site (XZ). Then *t*-test (between two treatments) was performed to disclose whether there existed differences among different treatments. It showed that Ascomycota had the significant differences (*P* = 0.031 < 0.05, **Figure 4B**).

**FIGURE 5:**
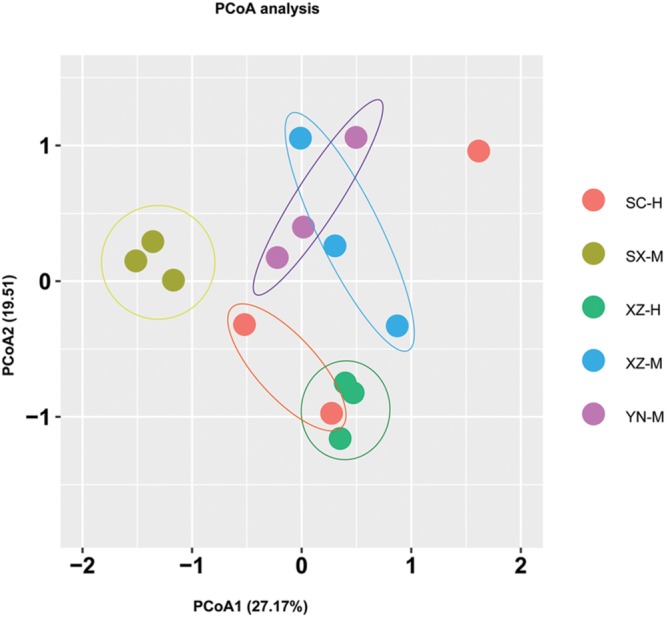
**Principal coordinate analysis (PCoA) of fungal community composition affected by site and altitude.** The PCoA analysis was conducted and plotted using packages of “vegan” and “ggplot2,” respectively, in R. Different color of circle dots represents the five sets samples, respectively. Three circle dots with same color mean the three duplicates of the same group of samples.

**FIGURE 6:**
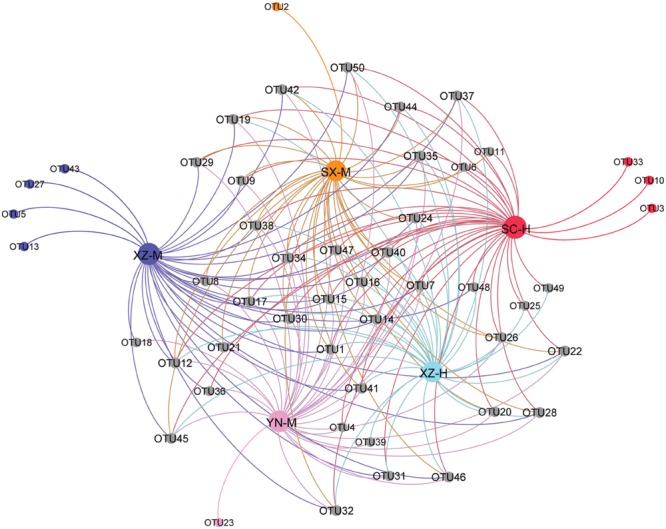
**Gehpi diagram of total 50 OTUs in the five sets samples.** Gephi 0.8.2 software was performed to illustrate the common and the special OTUs distribution among the 5 five sets samples. The gray circle dots represent the common OTUs, while the circle dots with different colors represent the special OTUs contained in the each group of samples.

From the PCoA (**Figure 5**), it can be seen that three duplicates of each-site samples had comparatively similar fungal community composition except one of the samples of SC-H, which happened the deviation with two axils, indicating the two factors corresponding to the two axils took obvious effect. Because the PCoA only clarified about 47% of the total OTUs, gehpi diagram (**Figure 6**) fully covering all the OTUs were shown here. **Figure 6** showed that more than half of the total OTUs shared among the samples, and several OTUs were site and altitude specific in the five sets of samples.

## Discussion

According to previous researches on lichen-associated fungal community, the target genes used for high-throughput sequencing were 18S rDNA and ITS (Bates et al., 2012; Park et al., 2015). In this study, we chose 18S rDNA as the target gene. The OTU number of lichen-related fungi in each sample of *H. hypotrypa* varied between 24 and 33, but the total number was 50, suggesting that there must be factors affecting the lichen-associated fungal community composition within *H. hypotrypa*. Two ecological factors (i.e., site and altitude) were analyzed on the correspondence to the different lichen-associated fungal community composition, and the results actually showed that both site and altitude took the significant effect within species at the OTU and class levels. Furthermore, six OTUs (OTU15, OTU34, OTU38, OTU40, OTU46, and OTU50) and two classes (Dothideomycetes and Sordariomycetes), and 1 phylum (Ascomycota) had been indicated the significant difference corresponding to site and altitude, respectively (**Figures 3** and **4**).

Of the 50 OTUs of lichen-associated fungi, including 4 phyla, 13 classes, 20 orders, 29 families and 25 genera, 56% (28 OTU) were the members of Ascomycota, 22% (11) of Basidiomycota, 8% (4) of Zygomycota, 6% (3) of Chytridiomycota, and 8% (4) of unknown fungi (Supplementary Table S3). These findings are consistent with those reported in other studies (U’Ren et al., 2014; Zhang et al., 2015). However, the Tibetan samples (XZ-H-3) including Basidiomycota as main fungal component, had XZ-H-1 and XZ-H-2 with near equal ratio of Basidiomycota as well as Ascomycota. At the phylum level, the percentage of lichen-associated fungal community composition was different among the 15 samples, and altitude was proved to be the significant factor. At the class and OTU level, both, site and altitude were resulted as significant factors. Further, within Ascomycota, four major classes (i.e., Dothideomycetes, Eurotiomycetes, Leotiomycetes, and Sordariomycetes) were included in the 15 samples (**Figure 2**), which were rather similar to those identified from the lichen species of temperate and Arctic regions (U’Ren et al., 2014; Zhang et al., 2015). However, these were different from the samples of Antarctic region (Park et al., 2015). Within Basidiomycota, one major class (Tremellomycetes) was identified (**Figure 2**), which was in part identical with other studies (Park et al., 2015; Zhang et al., 2015). The high proportion of Basidiomycota contained in the three samples of XZ-H showed lichen-associated fungal composition similar to the endophytes of plant root (Blaalid et al., 2014), but not to the endophytes of moss as previously reported (U’Ren et al., 2010, 2012), suggesting that high-altitude Tibet could be a special site for the lichen-associated fungal community composition. Further, our study highlight the importance of high-altitude Tibet areas to be further investigated for lichen-associated fungal community.

The correlation between lichen-associated fungi and some ecological factors had been discussed before. Higgins et al. (2014) reported that there was no evidence for host or habitat specificity in lichen-associated fungi, instead, spatial structure was proved to be important factor – i.e., distance between sites-significantly affected the lichen-associated fungal community composition. Endophytic fungal communities were also indicated changing with seasonal shift (Beck et al., 2014). Few studies dealt with the correlation between the lichen-associated fungal community composition and ecological factors. Harutyunyan et al. (2008) discussed lichen-associated black fungi isolated from 13 lichen species based on cultural strains, in which different altitudes, substrates and sites of lichen samples were considered. However, they focused more on the relationship between black fungi species and host, the effect of ecological factors such as altitude and site were not involved. U’Ren et al. (2012) comprehensively evaluated how the biotic, biogeographic and abiotic factors structured endophytic and endolichenic fungal communities also using the culture-based isolations. They concluded that ‘fungi from closely related hosts from different regions were similar in higher taxonomy, but differed at shallow taxonomic levels.’ Similarly, in our study site, i.e., different regions, significantly affected the lichen-associated fungal community composition at lower taxonomic level, i.e., OTU and class levels, but not at higher taxonomy level, i.e., phylum. However, that didn’t mean the higher taxonomy would be constant among the fungi from closely related hosts, for example, our study showed altitude had a significant effect on the lichen-associated fungal composition at phylum level, which had not been mentioned in the publication of U’Ren et al. (2012). Therefore, our study added more geographic factor like altitude to discuss the effect on the lichen-associated fungal community composition rather than climatic factors, and supplied more information of ecological analysis in detail. Nevertheless, there was a limitation that we only chose one lichen species as model and case study, and most of the differences at OTU, class and phylum levels among the samples were limited in the relative abundance of lichen-associated fungi, not the specific OTUs. Although, it indicated several OTUs were specific to the sites and altitudes from the gehpi diagrams, their reads numbers were rather low. Therefore, the question whether there existed specific OTUs corresponding to different sites and altitudes within lichen species remains open. Another defects was the altitude gradient, i.e., only two altitudes (M and H) were included in this research due to our incomplete investigation and filed work. Therefore, more lichen species, ecological factors and statistical analysis would be comprehensively considered and more objective results would be shown in the near future.

Functional contexts of lichen-associated fungi have almost been unexplored till now. Arnold et al. (2009) ever discussed about trophic transition networks for lichen-associated Ascomycetous fungi. It was found that endophytism can be transient evolutionarily, and lichens play an important role as cradles of symbiotroph diversification in Ascomycota. In particular, endolichenism seems like to be an evolutionary source for host shift among transitions to parasitic/pathogenic, saprotrophic, and especially endophytic states. The lichen-associated fungi in our study, for example, within Ascomycota, four major classes (i.e., Dothideomycetes, Eurotiomycetes, Leotiomycetes, and Sordariomycetes) were included that were rather similar to those from the temperate and Arctic lichen (U’Ren et al., 2014; Zhang et al., 2015), among which it has ever been reported that pathogen to endophyte transitions were especially common in Dothideomycetes and Sordariomycetes (Berbee, 2001). On the other hand, one major class of Basidiomycota (Tremellomycetes) was identified, which was rather abundant in the Tibetan lichen samples (XZ-H). OTU14 (*Tremella*), which belongs to Tremellomycetes, was reported isolated from extreme habitat (Lopez-Archilla et al., 2004). In addition, among 11 Basidiomycota OTUs in our study, OTU1 (*Sporobolomyces*) and OTU44 (*Cryptococcus*) were frequently found in the Arctic soils (Zalar and Gunde-Cimerman, 2014), OTU20 (*Bensingtonia*) and OTU30 (*Agrocybe*) were identified only from tropical habitats (Vaz et al., 2009; Tedersoo and Smith, 2013). This allows us to hypothesize that these Basidiomycota OTUs probably relate to the abiotic stress resistance. We didn’t verify the function of lichen-associated fungi, including Ascomycota and Basidiomycota, etc. in this study, due to the absence of large-scale sampling. Additional endolichenic fungi from diverse lichen species and endophytes from different types of plants should be investigated. But the difficulty will also lie in the inconstant endolichenic fungal composition in different lichen species, which is not like endolichenic bacteria with host specificity in some degree, having been studied the localization in lichen thallus and function through comparative omics approach (Grube et al., 2015). The function of lichen-associated fungi maybe the next research hotspots due to its important role as community component in this special symbiotic lifestyle following after the endolichenic bacteria.

## Conclusion

To answer the questions being put forward at the beginning: (1) there were some lichen-associated fungi detected in *H. hypotrypa* showed host-specific trait, although the lichen associated fungi from most *H. hypotrypa* samples were similar to those of other lichen species. Meanwhile, we also found that lichen-associated fungal community composition of *H. hypotrypa* samples collected from higher altitude in Tibetan Plateau (i.e., XZ-H) was similar to fungal component of plant, but not to moss, which was different from the conclusions being drawn in the previous studies; (2) our results showed that lichen-associated fungal community composition within *H. hypotrypa* was significantly shaped by both site and altitude at the OTU and class levels, whereas by altitude at phylum level, respectively.

## Author Contributions

YW collected the samples, extracted the DNA, analyzed the raw DNA data and prepared the tables and **Figures 1**, **2**, **5**, and **6**; YZ joined the statistical analyses, prepared **Figures 3** and **4** and revised the manuscript; XWa collected the samples and revised the manuscript; XWe designed this research, collected the samples, joined extracting the DNA and analyzing the raw DNA data, and wrote the main manuscript text; JW gave good advices on the manuscript. All authors reviewed the manuscript.

## Conflict of Interest Statement

The authors declare that the research was conducted in the absence of any commercial or financial relationships that could be construed as a potential conflict of interest.

## Footnotes

1 http://drive5.com/uparse/
